# Clinical characteristics, genomic profiling, treatments, and outcomes of Langerhans cell sarcoma

**DOI:** 10.1186/s13023-026-04199-4

**Published:** 2026-01-20

**Authors:** Min Lang, Xiao-juan Zheng, Long Chang, Dao-bin Zhou, Wei Zhang, Xin-xin Cao

**Affiliations:** 1 Department of Hematology, Peking Union Medical College Hospital, Chinese Academy of Medical Sciences & Peking Union Medical College, Beijing, China; 2 State Key Laboratory of Complex Severe and Rare Diseases, Peking Union Medical College Hospital, Chinese Academy of Medical Sciences & Peking Union Medical College, Beijing, China; 3 Department of Medical Oncology, National Cancer Center/National Clinical Research Center for Cancer/Cancer Hospital, Chinese Academy of Medical Sciences and Peking Union Medical College, Beijing, China

**Keywords:** Langerhans cell sarcoma, Target therapy, Prognosis

## Abstract

**Background:**

Langerhans cell sarcoma (LCS), an exceptionally rare and aggressive neoplasm, remains poorly characterized due to its scarcity. To address this knowledge gap, we conducted a retrospective analysis of 13 LCS patients. This retrospective study included patients ≥ 18 years old with biopsy proven LCS from October 2015 to April 2025.

**Results:**

The median age at diagnosis was 59 years (range: 33–71). The most commonly affected organs were the subcutaneous soft tissue (61.5%), followed by lymph nodes (53.8%), skin (30.8%), and bone (23.1%). *CBL* was the most common mutation, detected in four patients (33.3%). Notably, first-line treatment options included surgery and chemotherapy, with an overall response rate of 53.8%. Among all the relapsed or refractory patients, three eventually received targeted therapies (two trametinib and one niraparib), demonstrating promising efficacy with all patients achieved partial remission. With a median follow-up of 18.2 months (range: 2.6–93.1), the estimated 2-year overall survival rate was 92.3%, while the estimated 2-year progression-free survival (PFS) rate stood at 32.9%.

**Conclusions:**

In our cohort of LCS, we found that the PFS of LCS was poor. Genetic sequencing and the use of targeted therapies may offer a survival advantage for patients with LCS.

**Clinical trial registration:**

Not applicable.

**Supplementary Information:**

The online version contains supplementary material available at 10.1186/s13023-026-04199-4.

## Introduction

Langerhans cell sarcoma (LCS) is a rare malignant histiocytosis characterized by multi-organ involvement and an aggressive clinical course, classified under the M group in the Histiocyte Society classification of histiocytic disorders [1]. The overall incidence of the disease was 0.2 per 10,000,000 and did not differ by race or sex [2]. Histopathologically, LCS is identified by the presence of cytologically malignant histiocytoid cells with longitudinally grooved nuclei, prominent nucleoli, and a Langerhans cell phenotype by immunohistochemistry with expression of CD68, CD1a, and CD207 (Langerin). While LCS and Langerhans cell histiocytosis (LCH) share overlapping immunohistochemical profiles, diagnostic differentiation requires assessment of elevated Ki-67 proliferation index and cytologic atypia in LCS [3].

Due to lack of pathologically confirmed cases in the literature, there is a paucity of data in LCS. Little is known about the molecular data in people with LCS. One study of malignant histiocytosis (MH) included eight patients with LCS and identified genetic mutations, such as *KRAS,TP53,NRAS,BRAF,MAP2K1*, and *PTPN11* [4].

Since the existing treatment-specific information are derived from case reports and registry-based studies, the standard of treatment has not been established. There is currently no consensus on treatment options for LCS, surgery has a role in localized disease, treatments such as conventional lymphoma therapy protocols using the cyclophosphamide, doxorubicin, vincristine, and prednisone (CHOP) regimen and soft tissue sarcoma regimen (doxorubicin, ifosfamide, and dacarbazine) have been used, while further research is required to obtain a consensus on chemotherapeutic regimen. Targeted therapies, such as MEK inhibitors, have shown promising results in patients with specific genetic mutations [5].

Above all, to fill the knowledge gap of LCS, this study integrated clinical evaluation, molecular landscape profiling, and longitudinal outcome analysis in a LCS cohort of 13 patients.

## Methods

### Patients

This retrospective study included patients diagnosed with LCS from October 2015 to April 2025. We included patients ≥ 18 years old with biopsy proven LCS by two specialized pathologists in this analysis. The histological findings were consistent with LCS on the basis of the WHO classification of hematopoietic neoplasms [6]. The study was conducted in accordance with the ethical standards of the 1964 Declaration of Helsinki and its subsequent amendments. Ethical approval and waivers of informed consent were obtained from the Peking Union Medical College Hospital Ethics Committee and National Cancer Center/National Clinical Research Center for Cancer/Cancer Hospital.

### Data collection

In this study, patient demographics, disease characteristics and treatments were collected through electronic medical records. Baseline characteristics included the gender of the patient, age at diagnosis, disease onset location and clinical manifestations. Laboratory examination including complete blood count, liver and renal function. Radiological data, including thoracic and abdominal computed tomography (CT) and 18 F-fluorodeoxyglucose positron emission tomography (FDG-PET), were collected. Patients with available samples, tissues from the lesions underwent capture-based RNA sequencing for fusion gene and single nucleotide variant detection (Supplementary Table 1) or DNA target gene sequencing of 183 genes as previously described [7] (Supplementary Table 2).

### Treatment and outcomes

The treatment regimens included chemotherapy, targeted therapy and surgical interventions. Therapeutic response was assessed using PET Response Criteria in Solid Tumors (PERCIST 1.0) [8] or the Response Evaluation Criteria in Solid Tumors 1.1 (RECIST 1.1) [9], with outcomes categorized as complete response (CR), partial response (PR), stable disease (SD), or progressive disease (PD). Although not prospectively defined in the study protocol, all patients underwent appropriate clinical imaging (CT or FDG-PET) enabling retrospective application of these criteria to acquired scans and radiological reports. Patient outcomes were collected by clinical follow-up and telephone follow-up, and the last follow-up was on May 1, 2025. If the patient was not contacted at the last follow-up, we analyzed the patients’ conditions between diagnosis and the last follow-up record. Progression-free survival (PFS) was defined as the duration from diagnosis of LCS to the date of disease progression or death from any cause or last follow-up for patients with no recorded date of progression or death. Overall survival (OS) was defined as the duration from the diagnosis of LCS to the date of death from any cause or last follow-up.

### Statistical analysis

Descriptive statistics were used to summarize the demographic profile and disease characteristics of the patients. Categorical variables were compared using Fisher’s exact test, while continuous variables were compared with the Mann-Whitney U test. PFS and OS were estimated using the Kaplan-Meier method, with log-rank tests to compare differences between groups. Univariate and multivariate Cox regression models were used to compare variables of interest. *p* < 0.05 was considered statistically significant. Statistical analyses were performed using R software (version 3.6.3) and the R package ComplexHeatmap (RRID: SCR_017270), and SPSS 25.0 (SPSS Inc., Chicago, Illinois, USA, RRID: SCR_002865).

## Results

### Demographics and disease characteristics

The study included 13 patients with primary LCS, including 5 males and 8 females. The median age at diagnosis was 59 years (33–71), and the median duration from symptom onset to diagnosis was 16.9 months (1.0-152.9). All the patients underwent full body CT scans at diagnosis, and the proportion of patients underwent FDG-PET was 84.6% (*n* = 11). Clinical manifestations leading to diagnosis were dominated by mass causing symptoms (10 cases), with additional symptoms such as pain, itching, rash, loosening of tooth and arginine vasopressin deficiency presented in one patient each. The most commonly affected organs were the subcutaneous soft tissue (61.5%), followed by lymph nodes (53.8%), skin (30.8%), bone (23.1%), pituitary gland, spleen, oral mucosa and gum in one patient each. Five patients had a single lesion, with three cases affecting subcutaneous soft tissue, one case affecting the skin, and one affecting the gum; the remaining eight patients had multiple lesions. The Ki-67 index of the lesion tissues in all patients was ≥ 60%. The clinical and pathological characteristics of each patient are presented in Table 1.

**Table 1:** Table 1 Clinical characteristics of LCS patients

ID	Sex	Age (years)	Time to diagnosis (months)	Site of lesion	Ki-67 index	Genomic profiling	First-line treatment	Response evaluation	PFS (months)	Survival state	OS (months)
LCS-01	Female	71	2.2	Bone, subcutaneous soft tissue, lymph nodes, spleen	60%	KRAS、IGHD、KMT2D、TNFAIP3	CHOP	PD	2.6	Death	2.6
LCS-02	Male	61	22.1	Skin, lymph nodes	80%	CBL、PTPN11	CHOP、brentuximab vedotin、cladribine	PD	12.3	Survive	14.6
LCS-03	Female	59	24.0	Subcutaneous soft tissue	60%	Negative	Surgery	CR	79.3	Survive	79.3
LCS-04	Female	70	36.0	Subcutaneous soft tissue, lymph nodes	80%	CBL	Surgery	PD	76.7	Survive	93.1
LCS-05	Male	49	16.9	Gum	80%	Negative	Cytarabine monotherapy (1 g/m² q12h ×3d/cycle; 4 cycles)	PD	11.4	Survive	33.5
LCS-06	Female	64	86.3	Subcutaneous soft tissue, lymph nodes	80%	Negative	cladribine (5 mg/m²/d) plus cytarabine (1 g/m²/d ×5d/cycle) for 4 cycles	PR	7.3	Survive	7.3
LCS-07	Female	41	5.0	Subcutaneous soft tissue	80%	CBL、TP53	Cytarabine monotherapy (1 g/m² q12h ×3d/cycle; 4 cycles)	PR	10.1	Survive	10.1
LCS-08	Male	33	16.4	Bone, skin, subcutaneous soft tissue, lymph nodes	60%	SCN1A	cytarabine (100 mg/m² q12h ×7d/cycle) plus idarubicin (60 mg/m²/d ×3d/cycle) for 4 cycles	PR	11.7	Survive	11.7
LCS-09	Female	43	19.0	Skin, lymph nodes	60%	EZH2	CHOP	PD	4.6	Survive	6.9
LCS-10	Male	61	13.0	Bone, pituitary gland, oral mucosa	80%	Untested	cytarabine (100 mg/m²/d ×5d/cycle) with methotrexate (1 g/m²/d ×1d/cycle) for 6 cycles	CR	20.8	Survive	86.8
LCS-11	Male	37	2.2	Subcutaneous soft tissue	70%	KRAS、PTPN11、MAP2K1	cytarabine (100 mg/m² q12h ×7d/cycle) plus idarubicin (60 mg/m²/d ×3d/cycle) for 4 cycles	CR	43.9	Survive	43.9
LCS-12	Female	61	152.9	Skin	70%	TP53、ABL2、ARID2、BRCA2、CREBBP、DNMT3A、EZH2、FCGR2B、ATM::WNK2 fusion	CHOP	PD	10.8	Survive	25.0
LCS-13	Female	56	1.0	Subcutaneous soft tissue, lymph nodes	60%	CBL、TP53、PTPN11、NTRK3、CDH1、SPOP、TTF1、CHEK2、TSC1 deletion	Surgery, radiotherapy	CR	17.2	Survive	18.2

LCS: Langerhans cell sarcoma; OS: overall survival; PFS: progression-free survival; CHOP: cyclophosphamide, doxorubicin, vincristine, and prednisone; MTX: methotrexate; CR: complete response; PR: partial response; PD: progressive disease

### Genomic profiling

**Fig. 1:**
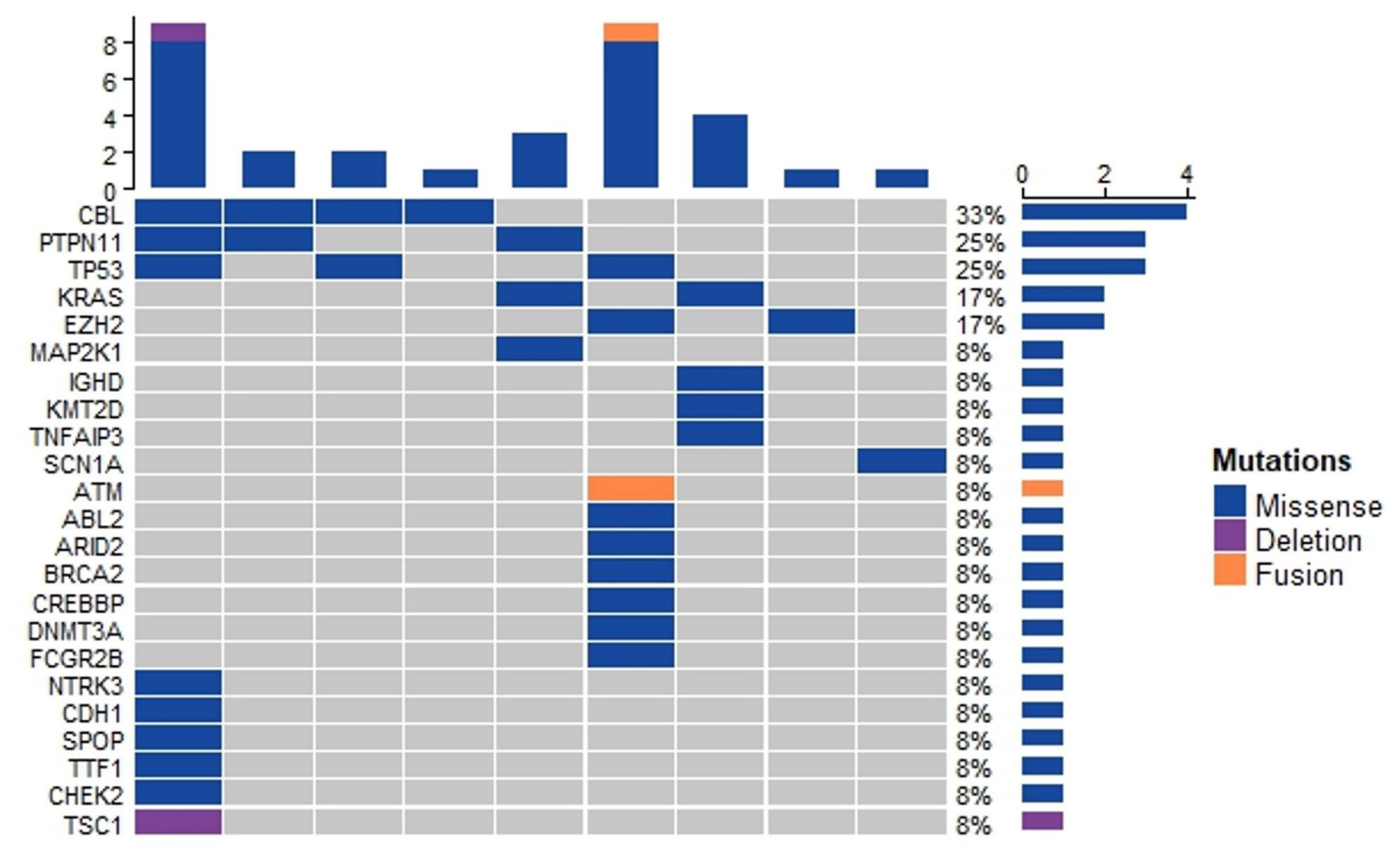
Genomic profiling of lesion tissues of LCS patients

Among 13 patients with LCS, targeted DNA sequencing of lesion tissues was successfully performed in 12 cases, supplemented by RNA sequencing in one case. Three patients (25.0%) showed no detectable alterations. The alterations were categorized into three groups: *MAPK/PI3K* pathway alterations (6/12, 50%), comprising recurrent *CBL* mutations (*n* = 4, 33.3%), *PTPN11* mutations (*n* = 3, 25.0%), *KRAS* mutations (*n* = 2, 16.7%), and single instances of *MAP2K1* mutation and *TSC1* deletion (each 8.3%); epigenetic regulator alterations, including *EZH2* mutations (*n* = 2, 16.7%) and single alterations in *KMT2D,ARID2,CREBBP* and *DNMT3A* (each 8.3%); and other alterations such as *TP53* mutations (*n* = 3, 25.0%) along with single cases of *IGHD,TNFAIP3,SCN1A,ABL2,BRCA2,FCGR2B,NTRK3,CDH1,TTF1,SPOP* and *CHEK2* mutations, plus one *ATM::WNK2* fusion (each 8.3%) (Fig. 1).

### Treatment and efficacy

**Fig. 2:**
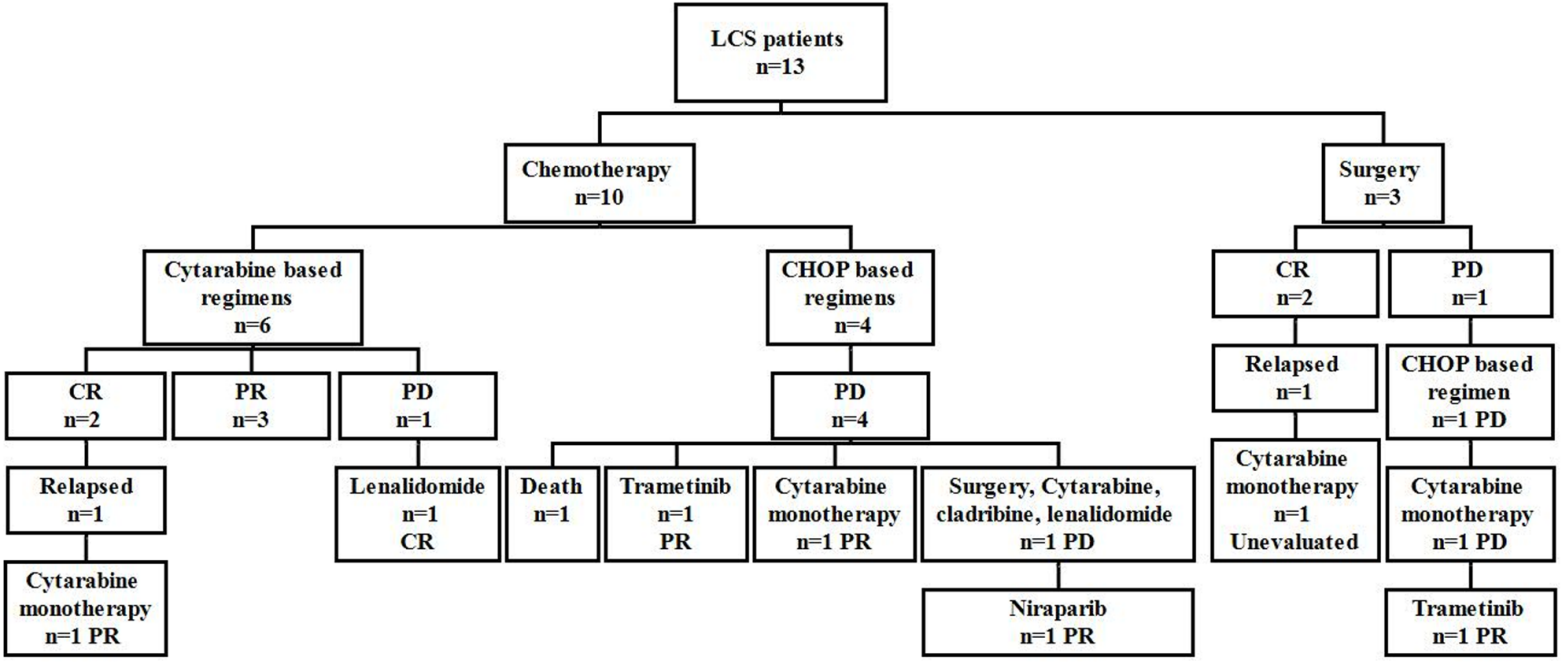
Treatment for the LCS patients. LCS: Langerhans cell sarcoma; CHOP: cyclophosphamide, doxorubicin, vincristine, and prednisone; CR: complete response; PR: partial response; PD: progressive disease

The treatment for the cohort is illustrated in a flow diagram in Fig. 2. Three patients underwent surgical removal of a single subcutaneous soft tissue lesion, and one of them also received lymph nodes radiotherapy after the operation. Other patients all received chemotherapy as initial treatment. Six patients received cytarabine based regimens, in which two received cytarabine monotherapy, two were combined with idarubicin, one was combined with methotrexate (MTX) and one with cladribine. Four patients received CHOP based treatment, including one patient with lesion expressing CD30 received CHOP in combination with brentuximab vedotin and cladribine.

**Fig. 3:**
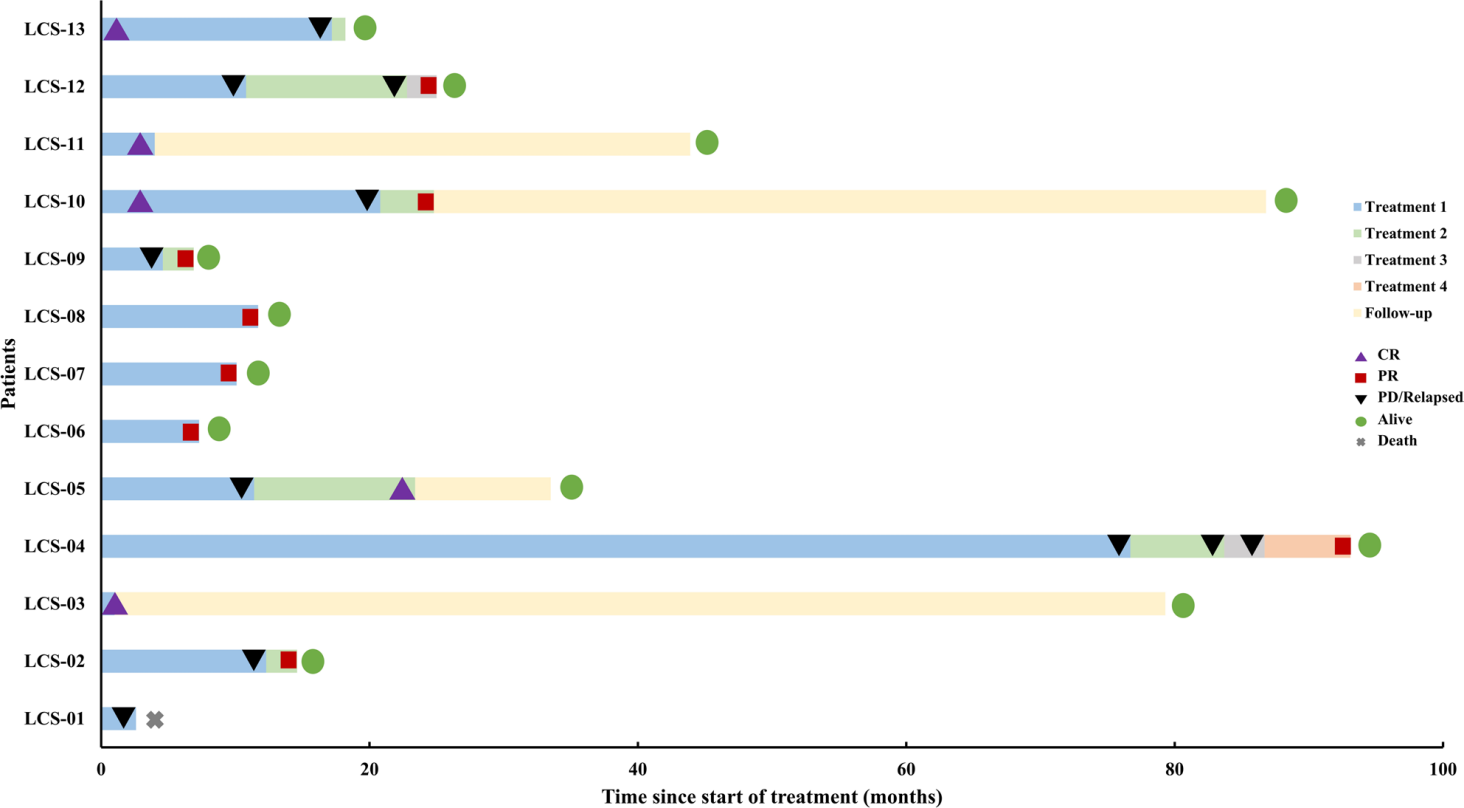
Treatment timelines and response durations for LCS patients

In this cohort of 13 patients, first-line therapy achieved an objective response rate (ORR) of 53.8%, comprising four CR and three PR. Among all the relapsed or refractory patients, three eventually received targeted therapies. Three patients received targeted therapy: two with MEK inhibitor trametinib (one with *CBL* mutation, the other with *CBL* and *PTPN11* mutations), and one with PARP inhibitor niraparib (harboring *BRCA2* mutation). All of them achieved PR, demonstrating promising efficacy (Fig. 3).

### Prognosis

**Fig. 4:**
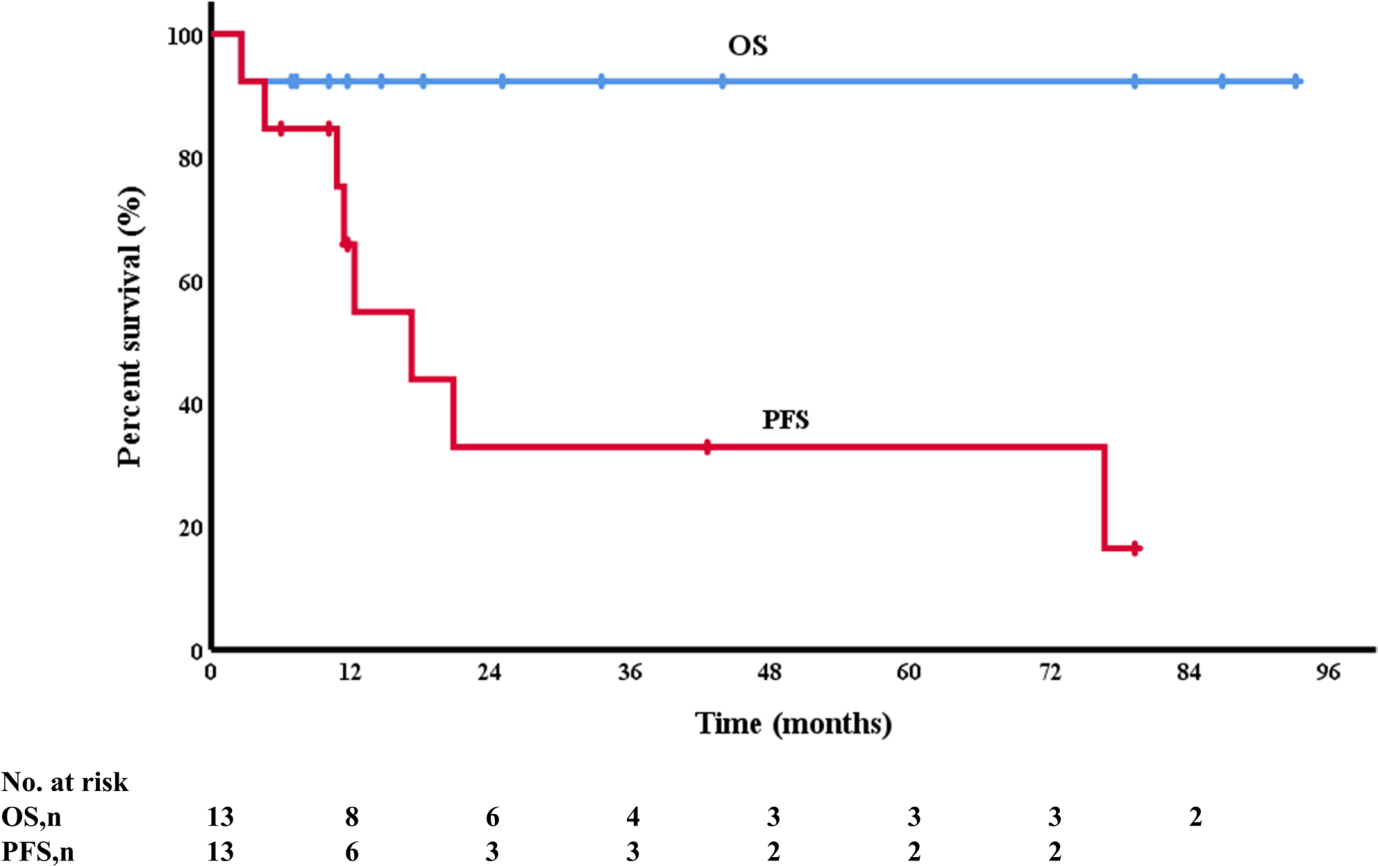
OS and PFS of the LCS patients

The median follow-up duration of the 13 patients was 18.2 months (range, 2.6–93.1 months). One patient died and the estimated 2-year OS rate was 92.3%. Totally 8 patients experienced disease progression during the follow-up process, with six progressing following first-line therapy and two relapsing after achieving CR to initial treatment. The estimated 2-year PFS rate was 32.9% (Fig. 4). Compared with patients who received other regimens for first-line treatment, patients who received CHOP based regimens for first-line treatment had a worse PFS. Median PFS was 7.7 months and 17.2 months for patients who received CHOP based regimens as first-line treatment and those who did not, respectively (*p* = 0.014, 95% CI 0.007–0.573). Among patients with single lesions, the 2-year OS and PFS rates were 100% and 50%, respectively; for those with multiple lesions, the rates were 87.5% and 18.8%. No significant difference in OS (*p* = 0.657) or PFS (*p* = 0.341) was observed between the groups.

## Discussion

In the Histiocyte Society classification and International Consensus Classification, MH encompasses histiocytic sarcoma, LCS, and interdigitating dendritic cell sarcoma. Patients with LCS may present with multi-organ involvement including skin, lymph nodes, bone, lung, bone marrow, and brain, and previous report showed that lymph nodes and skin were the most common organs involved [10]. Similarly, the most commonly affected organs were the subcutaneous soft tissue and lymph nodes in our research.

Notably, LCS exhibits a distinct molecular landscape characterized by recurrent somatic mutations and genomic instability, contrasting with the predominantly *BRAF*^*V600E*^ driven pathogenesis of LCH [11]. A genomic study of ten LCS patients revealed recurrent alterations in *CDKN2A* (50%), *TP53* (40%), *KRAS* (40%), *PTEN* (30%), *BCL2* (20%), *BRAF*^*V600E*^ (10%), and *CSF1R* (10%) [12]. In our research, 92.3% (12/13) of the patients underwent genetic testing, which is the cohort with the largest number of genetic tests conducted on LCS patients to date. *CBL* mutations were detected in 33.3% patients, followed by *TP53* and *PTPN11* each in 25.0%. *CBL* encodes multifunctional adapter proteins that regulate signal transduction in physiological and pathological processes, particularly pathways implicated in oncogenesis, hematopoiesis, and T-cell receptor regulation. While *CBL* mutations have been documented in LCH [11], juvenile myelomonocytic leukemia [13], primary myelofibrosis [14] and systemic mastocytosis [15], their recurrent occurrence in LCS (33.3% of cases) represents a novel finding. This suggests potential dysregulation of *MAPK/PI3K* signaling in a subset of LCS. The *ATM::WNK2* fusion identified in our study represents a novel finding in LCS, with no prior reports of this fusion in any malignancy. While somatic mutations in *ATM* and *WNK2* have been documented separately in Waldenström macroglobulinemia [16] and gastric cancer [17], their fusion configuration is unreported. Functionally, *ATM* encodes a master kinase governing DNA damage response, whereas *WNK2* regulates ion transport through serine/threonine kinase activity. The functional consequences of this fusion and its potential as a therapeutic target warrant further investigation. The mutation lineages of LCH and LCS were different, which underscored the necessity for comprehensive molecular profiling in suspected LCS cases to confirm diagnosis and guide therapeutic decisions.

Owing to its rarity, standard treatment for LCS has not been established to date. Of the 52 LCS patients from NCDB, 20 received chemotherapy as first-line therapy, 24 received surgery, and 15 received radiotherapy. Patients who were managed with radiotherapy had a better OS compared to those who received no radiotherapy [2]. Another study reviewed 46 case series including 66 subjects with LCS, chemotherapy was used alone or in combination in 47 cases (71%) followed by surgery in 31 cases (47%) [5]. Bendamustine [18], EPOCH (etoposide, prednisone, vincristine, cyclophosphamide, and doxorubicin) and pegylated interferon-α2b [19] regimens have also been used in patients with LCS [20]. *BRAF*^*V600E*^ mutated LCS patients can get temporary remission under treatment with BRAF inhibitor dabrafenib [21]. In our study, most patients are treated with cytarabine based regimen, and the vast majority of patients treated with this regimen in the first and second line can achieve disease remission. However, patients receiving the CHOP based regimen all progressed. The salvage therapies of trametinib and niraparib obtained promising therapeutic effect. Given its high-risk nature, management of LCS warrants aggressive therapeutic strategies, especially molecularly targeted agents. Early integration of next-generation sequencing combined with RNA sequencing and exploration of novel targeted therapies are strongly recommended to improve survival outcomes in this rare malignancy. In our cohort, two LCS patients harbored *EZH2* mutations. Although *EZH2* inhibitors are not currently approved for clinical use, preclinical evaluation of their therapeutic potential in LCS is warranted.

National database studies from the United States have reported a median OS of 19 months for LCS, and the 1-year OS rate was 62% [2].The prognosis observed in our cohort appears more favorable than historical reports, with an estimated 2-year OS rate of 92.3%. This potentially improved survival may be attributed to several factors: exclusion of secondary LCS cases, systematic molecular profiling, utilization of targeted therapies in relapsed/refractory settings, and the relatively small sample size with inherent selection bias characteristic of retrospective single-center studies. Previous studies have shown that in MH, whether the lesion is single or multiple varies significantly in prognosis [4]. Another systematic review of LCS also concluded that localized lesions are curable with surgery [5]. However, in this cohort of LCS, no significant difference in OS or PFS was observed between the groups.

In our cohort of LCS, we found that the PFS of LCS was poor. This study advances our understanding of LCS by delineating its distinct clinical and molecular landscape and therapeutic vulnerabilities. The historically poor overall survival observed in LCS underscores the critical importance of comprehensive molecular profiling, including both DNA-based NGS and RNA sequencing, to identify actionable targets for precision therapy. Notably, the relatively favorable outcomes in our cohort may reflect the early adoption of molecularly guided interventions, as patients failing first-line therapies frequently received targeted agents tailored to their mutational profiles. However, further validation in larger, multicenter studies is required. Future research should focus on optimizing molecularly targeted therapies and individualized treatment strategies to improve survival rates and quality of life for LCS patients.

## Conclusion

This study showed that LCS is extremely rare. Genetic sequencing and the use of targeted therapies may offer a survival advantage for patients with LCS.

## Supplementary Information

Below is the link to the electronic supplementary material.

Supplementary Material 1

## Data Availability

The data analyzed during the current study is available from the corresponding author upon reasonable request.

## Abbreviations

LCS: Langerhans cell sarcoma
WHO: World Health Organization
LCH: Langerhans cell histiocytosis
MH: Malignant histiocytosis
CHOP: Cyclophosphamide, doxorubicin, vincristine, and prednisone
CT: Computed tomography
FDG-PET: Fluorodeoxyglucose positron emission tomography
CR: Complete response
PR: Partial response
SD: Stable disease
PD: Progressive disease
PFS: Progression-free survival
OS: Overall survival
MTX: Methotrexate
ORR: Objective response rate
EPOCH: Etoposide, prednisone, vincristine, cyclophosphamide, and doxorubicin
