# Supplementary material for: Clinical characteristics, genomic profiling, treatments, and outcomes of Langerhans cell sarcoma
Source: Orphanet J Rare Dis. 2026 Jan 20;21:58. doi: 10.1186/s13023-026-04199-4 (PMC12903575; doi:10.1186/s13023-026-04199-4)
Supplement: Supplementary file 1 — Supplementary Material 1 [file 13023_2026_4199_MOESM1_ESM.docx]

| **List of fusion genes** | | | | | |
| --- | --- | --- | --- | --- | --- |
| **Name** | **Typical fusion** | **Name** | **Typical fusion** | **Name** | **Typical fusion** |
| ABCB1 | GRM3::ABCB1 | HDAC9 | HDAC9::MCMBP | PML | PML::RARA |
| ABL1 | ABL1::BCR | HINT2 | HINT1::DYNC2H1 | POGZ | POGZ::CHCHD3 |
| ABL2 | ABL2::ETV6 | HIST1H3B | HIST1H3B:: | POU5F1 | POU5F1::EWSR1 |
| ACTB | ACTB::GLI1 | HMGA2 | HMGA2::EBF1 | PPFIBP1 | PPFIBP1::ALK |
| ACVR1 | ACVR1::SGCD | HOXA9 | HOXA9::NUP98 | PPP1R36 | PPP1R36:: |
| AFF1 | AFF1::KMT2A | HSP90AB1 | HSP90AB1::DNAJC21 | PQLC1 | PQLC1:: |
| AFF3 | AFF3::RUNX1 | IDH1 | IDH1::NCL | PRC1 | PRC1::RCCD1 |
| AKT3 | AKT3::PVT1 | IDH2 | IDH2:: | PRPF40B | PRPF40B::DAZAP1 |
| ALK | ALK::EML4 | IKZF1 | IKZF1::BCL6 | PRPF8 | PRPF8::LDHA |
| APC | APC::RBMS1 | IL2 | IL2::TNFRSF17 | PRPS1 | PRPS1::PRKCB |
| ASPSCR1 | ASPSCR1::TFE3 | IQGAP2 | IQGAP2::PDGFB | PRSS3 | ZCCHC7::PRSS3 |
| ASXL1 | ASXL1::FTX | IRF4 | STPG1::IRF4 | PSMB5 | PSMB5:: |
| ASXL2 | KAT6A::ASXL2 | ITK | ITK::SYK | PTCH1 | PTCH1::LUC7L2 |
| ATF1 | ATF1::FUS | JAK2 | JAK2::BCR | PTK2B | EPHX2::PTK2B |
| ATM | ATM::ASPH | JAZF1 | JAZF1::CTNNA1 | PTPN11 | HDAC5::PTPN11 |
| AXIN1 | AXIN1::HDAC8 | KAT6A | KAT6A::CREBBP | RAF1 | RAF1::ACTR2 |
| AXIN2 | AXIN2::CFDP1 | KAT6B | KAT6B::FGFR1 | RANBP17 | RANBP17::TRD |
| BCL11B | BCL11B::TLX3 | KDM4C | KDM4C::JAK2 | RARA | RARA::NUMA1 |
| BCL2 | AFF3::BCL2 | KDM6A | KDM6A::ANOS1 | RB1 | RB1::CNIH3 |
| BCL6 | ARID4B::BCL6 | KDR | KDR::PDGFRA | RECK | REC::ALX3 |
| BCOR | BCOR::RARA | KIAA1549 | KIAA1549::BRAF | RELA | ACTB::RELA |
| BCR | BCR::ABL1 | KIT | KIT::UGT2B7 | RET | RET::NTRK1 |
| BIRC3 | BIRC3::MALT1 | KLK2 | KLK2::ETV4 | ROS1 | ROS1::CCDC6 |
| BRAF | BRAF::KIAA1549 | KMT2A | KMT2A::ABI1 | RPL21 | RPL21::ELF3 |
| BRCA1 | BRCA1::NFYB | KMT2B | KMT2B::AFF1 | RPN1 | RPN1::MECOM |
| BRCA2 | BRCA2::N4BP2L2 | KMT2C | KMT2C::ACTR3B | RPRD1B | RPRD1B::ZMYM4 |
| C17ORF50 | C17ORF50:: | KMT2D | KMT2D::RHEBL1 | RUNX1 | RUNX1::RUNX1T1 |
| CALR | CALR:: | KRAS | KRAS::KMT2A | RUNX1T1 | RUNX1T1::STXBP5 |
| CAMTA1 | CAMTA1::WWTR1 | LEUTX | KAT6A::LEUTX | SBDS | SBDS::POLE3 |
| CAPRIN1 | CAPRIN1::PDGFRB | LMNA | LMNA::ALK | SETBP1 | SETBP1::NUP98 |
| CBFA2T3 | CBFA2T3::RUNX1 | LMO1 | LMO1::SPACA6 | SF3A1 | SF3A1::PEA15 |
| CBFB | CBFB::MYH11 | LMO2 | LMO2::TRD | SF3B1 | NCOR1::SF3B1 |
| CBL | CBL::KMT2A | MAF | JCHAIN::MAF | SH2D1A | SH2D1A::TRA |
| CCDC6 | CCDC6::RET | MAFB | NSD2::MAFB | SLC45A3 | SLC45A3::ERG |
| CCL21 | CCL21::GEMIN5 | MAML2 | MAML2::CRTC1 | SMAD5 | SMAD5:: |
| CCNB3 | BCOR::CCNB3 | MAML3 | MAML3::SERPINE2 | SMARCA4 | SMARCA4::TYK2 |
| CCND1 | CCND1::NFAT5 | MAN2B1 | MAN2B1::RNASEH2A | SMARCA5 | SMARCA5::APPBP2 |
| CCND2 | CCND2::IGL | MAP2K2 | MAP2K2::CAMKK1 | SMARCB1 | SMARCB1::WASF2 |
| CCND3 | CCND3::IGH | MCM3AP | PTPRG::MCM3AP | SNX29 | SNX29::TNFRSF17 |
| CCR5 | CCR5:: | MECOM | MECOM::ETV6 | SOCS2 | WTAP::SOCS2 |
| CCR7 | SYNRG::CCR7 | MEF2B | MEF2B::BORCS8 | SP3 | SP3::TRPM3 |
| CDK1 | CDK1::USP24 | MEF2D | MEF2D::HNRNPUL1 | SRSF2 | SRSF2:: |
| CDK4 | CCND1::CDK4 | MET | MET::DYNC1I1 | SS18 | SS18::MEF2D |
| CEBPA | CEBPA:: | MKL1 | MKL1::PBX1 | SSBP2 | SSBP2::JAK2 |
| CHD7 | CHD7::TACC1 | MKL2 | MKL2:: | SSX1 | SSX1::SS18 |
| CIC | CIC::DUX4 | MLLT1 | MLLT1::KMT2A | SSX2 | SSX2::SS18 |
| CIITA | CIITA::BCL6 | MLLT10 | MLLT10::CDK6 | SSX4 | SSX4::SS18 |
| COL1A1 | COL1A1::PDGFB | MLLT3 | PAX5::MLLT3 | STAG2 | STAG2::XIAP |
| CREB1 | CREB1::EWSR1 | MLLT4 | AFDN::KMT2A | STAT3 | RARA::STAT3 |
| CREB3L1 | CREB3L1::FUS | MN1 | MN1::ETV6 | STAT5B | STAT5B::RARA |
| CREB3L2 | CREB3L2::IKZF1 | MPL | MPL:: | STAT6 | STAT6::ADGRL2 |
| CREBBP | CREBBP::KAT6B | MSI2 | MSI2::HOXA9 | TACC1 | TACC1::PLXNB2 |
| CRLF1 | ELL::CRLF1 | MYB | MYB::ESR1 | TAF15 | TAF15::ZNF384 |
| CRLF2 | CSF2RA::CRLF2 | MYBL1 | BCL6::MYBL1 | TAL1 | TAL1::STIL |
| CSF1R | SSBP2::CSF1R | MYC | MYC::ZCCHC7 | TBX21 | TBX21:: |
| CSF3R | CSF3R:: | MYH11 | MYH11::CBFB | TCF12 | TCF12::NR4A3 |
| CTBP1 | CTBP1::ATF6 | MYH9 | MYH9::ALK | TCF3 | TCF3::PBX1 |
| CTNNB1 | CTNNB1::AKAP11 | MYLK | MYLK::SIAE | TCL6 | TCL6::TCL1B |
| CUL2 | CCNY::CUL2 | MYO18A | MYO18A::FGFR1 | TERT | TERT::ALK |
| DAXX | EGR1::DAXX | MYOD1 | MYOD1:: | TET2 | TET2:: |
| DDIT3 | DDIT3::EWSR1 | NACA | NACA::BCL6 | TFCP2 | TFCP2:: |
| DICER1 | DICER1::CCDC14 | NCOA1 | PAX3::NCOA1 | TFE3 | TFE3::ASPSCR1 |
| DNMT3A | DNMT3A::NUMA1 | NCOA2 | NCOA2::KAT6A | TFG | TFG::NR4A3 |
| DNTT | DNTT:: | NCOR1 | NCOR1::FBXO3 | TGFB1 | TGFB1::HNRNPUL1 |
| DUSP22 | IRF4::DUSP22 | NDUFA4 | NDUFA4::RTN3 | TGIF1 | ANKRD11::TGIF1 |
| DUX4 | CIC::DUX4 | NF1 | NF1::EVI2A | THADA | THADA::EML4 |
| EGFR | EGFR::ACADM | NFATC2 | NFATC2::RAE1 | THRA | THRA::B4GALNT2 |
| ELL | ELL::KMT2A | NFE2L2 | NFE2L2::NACA | TLX1 | TLX1::TRA |
| EML4 | EML4::ALK | NFKB2 | NFKB2::TBXAS1 | TLX3 | TLX3::BCL11B |
| EP300 | EP300::BCOR | NFYC | NFYC::RPLP2 | TMEM2 | TMEM2:: |
| EPC1 | EPC1::ZEB1 | NKD2 | EHBP1::NKD2 | TMEM259 | TMEM259::FASN |
| EPOR | EPOR::SFTPB | NOC4L | NOC4L::RAB3GAP2 | TMPRSS2 | TMPRSS2::ETV1 |
| ERBB2 | ERBB2::MAD2L2 | NOTCH1 | NOTCH1::NUP214 | TNFSF18 | RFWD2::TNFSF18 |
| ERG | ERG::DUX4 | NOTCH2 | NOTCH2::MNDA | TNRC18 | KMT2A::TNRC18 |
| ESR1 | ESR1::CCDC170 | NOTCH4 | CNNM2::NOTCH4 | TP53 | TP53::NTRK1 |
| ETV1 | ETV1::EWSR1 | NPM1 | NPM1::ALK | TP63 | TP63::TBL1XR1 |
| ETV4 | ETV4::TMPRSS2 | NR4A3 | NR4A3::ABL2 | TPM3 | TPM3::ALK |
| ETV6 (TEL) | ETV6::ABL1 | NRAS | SS18::NRAS | TRPS1 | TRPS1:: |
| EVI2A | NF1::EVI2A | NTRK1 | NTRK1::TFG | TSC1 | TSC1::CEL |
| EVI2B | EVI2B:: | NTRK2 | NTRK2::RASEF | TSC2 | TSC2::CHTF18 |
| EWSR1 | EWSR1::CREB1 | NTRK3 | NTRK3::ETV6 | TSLP | TSLP:: |
| EZH2 | EZH2::ATP2A2 | NUMA1 | NUMA1::RARA | TSPAN4 | TSPAN4::EPS8L2 |
| FAS | FAS::CTNND2 | NUP214 | NUP214::ABL1 | TYW1 | TYW1::HGSNAT |
| FASLG | PRRC2C::FASLG | NUP98 | NUP98::HOXA11 | U2AF2 | U2AF2::ECSCR |
| FBXW7 | FBXW7::ARHGAP10 | NUTM1 | NSD3::NUTM1 | UBTF | UBTF::ETV4 |
| FEV | FEV::FUS | NUTM2A | NUTM2A::YWHAE | USP6 | USP6::COL1A1 |
| FGFR1 | FGFR1::BCR | NUTM2B | NUTM2B::YWHAE | VCP | VCP::HNRNPA2B1 |
| FGFR2 | FGFR2::AFF3 | PALM | PIP5K1C::PALM | WHSC1 | NSD2::FOXP1 |
| FGFR3 | FGFR3::NSD2 | PATZ1 | PATZ1::EWSR1 | WT1 | EWSR1::WT1 |
| FIP1L1 | FIP1L1::PDGFRA | PAX3 | PAX3::FOXO1 | WWTR1 | WWTR1::FOSB |
| FLI1 | FLI1::EWSR1 | PAX5 | PAX5::CBFA2T3 | XIAP | XIAP:: |
| FLT3 | FLT3::ETV6 | PAX7 | PAX7::FOXO1 | YAP1 | YAP1::TFE3 |
| FOSB | SERPINE1::FOSB | PBX1 | PBX1::KMT2A | YY1 | YY1::EWSR1 |
| FOXO1 | FOXO1::PAX3 | PBX3 | PBX3::KDM5A | ZBTB18 | N4BP2L1::ZBTB18 |
| FOXO4 (AFX) | FOXO4::KMT2A | PCM1 | PCM1::JAK2 | ZC3H7A | ZC3H7A::PLCB4 |
| FOXP1 | FOXP1::ETV1 | PDGFB | PDGFB::COL1A1 | ZEB2 | ZEB2::JCHAIN |
| FUS (TLS) | FUS::ERG | PDGFRA | PDGFRA::FIP1L1 | ZFAND3 | ZFAND3::TBC1D5 |
| FYTTD1 | FYTTD1::DNAJC7 | PDGFRB | PDGFRB::ETV6 | ZMIZ1 | ZMIZ1::ABL1 |
| GATAD2A | GATAD2A::PRKACA | PHF6 | PHF6::STX12 | ZMYM3 | ZMYM3:: |
| GLIS2 | CBFA2T3::GLIS2 | PIK3CA | TBL1XR1::PIK3CA | ZMYND11 | ZMYND11::ZFAND4 |
| GNA11 | GNA11::REG1A | PIK3R1 | PIK3R1::KANK1 | ZNF362 | ZNF362::CEP250 |
| GNAQ | GNAQ::CELF2 | PIM3 | PIM3::CRELD2 | ZNF384 | ZNF384::TAF15 |
| GRIP1 | GRIP1::TBL1XR1 | PITX2 | PITX2::PPP1R15A | ZNF444 | EWSR1::ZNF444 |
| H3F3A | H3F3A:: | PLAG1 | PLAG1::CTNNB1 | ZRSR2 | ZRSR2:: |
| **List of single nucleotide variants** | | | | | |
| ABCB1 | ABL1 | ABL2 | ACTB | AKT3 | ALK |
| APC | ASXL1 | ASXL2 | ATM | AXIN1 | BCL11B |
| BCL2 | BCL6 | BCOR | BCR | BIRC3 | BRAF |
| BRCA1 | BRCA2 | CALR | CBFA2T3 | CBFB | CBL |
| CCND1 | CCND2 | CCND3 | CCR7 | CDK4 | CEBPA |
| CIC | CIITA | COL1A1 | CREBBP | CRLF2 | CSF1R |
| CSF3R | DICER1 | DNMT3A | DUSP22 | EGFR | EP300 |
| EPOR | ERBB2 | ERG | ETV6 | EWSR1 | EZH2 |
| FAS | FBXW7 | FGFR1 | FGFR2 | FGFR3 | FLT3 |
| FOXO1 | FOXP1 | HOXA9 | IDH1 | IDH2 | IKZF1 |
| IRF4 | ITK | JAK2 | KDM6A | KIT | KMT2A |
| KMT2B | KMT2C | KMT2D | KRAS | LMO2 | MAF |
| MAFB | MAML2 | MAP2K2 | MECOM | MEF2B | MET |
| MLLT1 | MLLT10 | MLLT3 | MPL | MYB | MYC |
| MYH11 | NCOR1 | NF1 | NFKB2 | NOTCH1 | NOTCH2 |
| NOTCH4 | NPM1 | NRAS | NTRK1 | NTRK2 | NTRK3 |
| NUP214 | NUP98 | PAX5 | PBX1 | PCM1 | PDGFRA |
| PDGFRB | PHF6 | PIK3CA | PIK3R1 | PIM3 | PML |
| POU5F1 | PRPF40B | PRPF8 | PRPS1 | PSMB5 | PTK2B |
| PTPN11 | RAF1 | RARA | RB1 | RET | ROS1 |
| RUNX1 | SBDS | SETBP1 | SF3A1 | SF3B1 | SH2D1A |
| SMARCA4 | SMARCB1 | SRSF2 | STAG2 | STAT3 | STAT5B |
| STAT6 | TAL1 | TCF12 | TCF3 | TERT | TET2 |
| TP53 | TP63 | TSC1 | TSC2 | U2AF2 | WHSC1 |
| WT1 | XIAP | YY1 | ZMYM3 | ZNF384 | ZRSR2 |

**Supplementary Table1: Fusion genes and single nucleotide variants for RNA sequencing**

| ALK | ANKRD50 | ANO10 | AP3B1 | ARAF | ARID1B |
| --- | --- | --- | --- | --- | --- |
| ASXL1 | ATM | ATP4A | ATP9A | ATRX | B2M |
| BCL1 | BCL2L1 | BCOR | BCORL1 | BIRC3 | BOB |
| BRAF | BRD4 | CALR | CASK | CBL | CCDC168 |
| CCR7 | CD274 | CDC73 | CDH1 | CDK6 | CDKN1A |
| CDKN2A | CDKN2B | CEBPA | CEP85 | CHEK2 | CHMP1A |
| COL6A6 | CSF1R | CSF3R | CTNNB1 | DKK2 | DNAH7 |
| DNMT3A | DUSP4 | ECE1 | EFNA4 | EGFR | EMSY |
| EOMES | ERBB2 | ERBB3 | ERBB4 | ETV6 | EZH2 |
| FAS | FBXW7 | FCGBP | FGFR2 | FGFR3 | FLT3 |
| FOXP3 | GATA1 | GATA2 | GNAQ | GNAS | GREB1L |
| GRK4 | HLA-DQA1 | HLA-DQB2 | HNF1A | HRAS | IDH1 |
| IDH2 | ING1 | ITIH6 | JAK1 | JAK2 | KDM5A |
| KDM6A | KDR | KIF5B | KIR2DL4 | KIT | KMT2B |
| KMT2D | KMT2E | KRAS | LYST | MAP2K1 | MAP3K1 |
| MAPK1 | MAPK8 | MAPK14 | MDM2 | MDM4 | MET |
| MLH1 | MSH2 | MSH6 | MUNC13-4 | MYC | MYD88 |
| NCOA2 | NCOA4 | NCOR2 | NEMO | NF1 | NFKB1 |
| NOTCH1 | NOTCH2 | NRAS | NT5DC3 | NTRK1 | PAX5 |
| PBRM1 | PBX1 | PCDHA8 | PDCD1 | PDCD1LG2 | PDCD2 |
| PDGFRA | PHIP | PICK1 | PIK3CA | PIK3CD | PIK3R2 |
| PKNOX2 | PLXNA2 | PMS2 | PNISR | POLD1 | POLE |
| PRF1 | PTEN | PTPN11 | RAB27A | RAF1 | RB1 |
| RBM10 | RET | RICTOR | ROS1 | RPS6 | RUNX1 |
| RYR2 | SCN1A | SEC62 | SETBP1 | SETD2 | SF3B1 |
| SH2D1A | SHOC2 | SLC10A6 | SLC29A3 | SMAD6 | SMPD1 |
| SOS1 | SPRED1 | SREBF1 | SRSF2 | ST8SIA1 | STAG2 |
| STAT3 | STK11 | STX11 | STXBP2 | TAP2 | TERT |
| TET2 | TLR7 | TLR8 | TP53 | TRBV20OR9-2 | TSC1 |
| TTN | U2AF1 | UNC13B | UNC13D | VCL | VEGFA |
| WT1 | XIAP | ZRSR2 |  |  |  |

**Supplementary Table2: 183 candidate genes for DNA target sequencing**
